# Regenerate bone stimulation following limb lengthening: a meta-analysis

**DOI:** 10.1186/s12891-016-1259-5

**Published:** 2016-09-29

**Authors:** Julio J. Jauregui, Anthony V. Ventimiglia, Preston W. Grieco, David B. Frumberg, John E. Herzenberg

**Affiliations:** 1 Department of Orthopaedics, University of Maryland Medical Center, 110 S. Paca Street, 6th Floor, Suite 300, Baltimore, Maryland 21201 USA; 2 SUNY Downstate Medical Center, Department of Orthopaedic Surgery and Rehabilitation, 450 Clarkson Avenue, Brooklyn, New York 11203 USA; 3 Rubin Institute for Advanced Orthopedics, Sinai Hospital of Baltimore, 2401 West Belvedere Avenue, Baltimore, Maryland 21215 USA

**Keywords:** Distraction, Osteogenesis, Fixation, Regenerate, Ultrasound, Limb, Bone Lengthening

## Abstract

**Background:**

Limb lengthening with external fixation is performed to treat patients with leg length discrepancy or short stature. Although the procedure has a high rate of success, one potential drawback from limb lengthening is the amount of time spent in the fixation device while regenerate bone consolidates. Although some studies have assessed different treatment modalities, there has not been a study that has systematically evaluated whether low intensity pulsed ultrasound (LIPUS) or pulsed electromagnetic fields (PEMF) have significant effects on regenerate bone growth. The purpose of this study was to evaluate these two non-pharmacological treatment options to stimulate regenerate bone, and to assess whether they affect the treatment time in limb lengthening.

**Methods:**

Utilizing the electronic databases Medline, Embase and Ovid, we performed a literature search for studies describing the application of LIPUS or PEMF following limb lengthening. With the aid of a statistical software package, Forest-Plots were generated to compare the differences in bone healing index with and without the use of regenerate bone stimulation.

**Results:**

A total of 7 studies assessed these two bone stimulation modalities in a cohort of 153 patients. Overall, the mean healing index was 11.7 days/cm faster when using bone stimulation that in the comparison cohorts (33.7 vs 45.4 day, standardized mean difference of 1.16; *p =* 0.003).

**Conclusion:**

Amongst the drawbacks from limb lengthening is the relatively high rate of non- and delayed-union. Several methods, both pharmacological and non-pharmacological, have been investigated for their potential to stimulate the growth of regenerate bone. After systematically evaluating the limited and heterogeneous current literature, we found that LIPUS and PEMF both decreased the time for bone healing (healing index in days/cm) of the newly formed regenerate bone in an adequately selected cohort of patients that underwent limb lengthening. However, a high number of complications should be noted, which could be attributed to the lengthening procedure or to the additional bone stimulation.

**PROSPERO registration number:**

CRD42016039024

**Electronic supplementary material:**

The online version of this article (doi:10.1186/s12891-016-1259-5) contains supplementary material, which is available to authorized users.

## Background

Leg length discrepancy and short stature have multifactorial etiologies that may include congenital, developmental, infectious, or posttraumatic. Limb lengthening is an acceptable option to manage patients with these conditions [1–5]. First described by Alessandro Codivilla in the early 20th century [6], this procedure gained popularity following the work of Gavriil Ilizarov and his development of the external fixation device [7]. Advances in the field since then have led to a wider array of methods with which to lengthen bone. Currently, limb lengthening can be achieved by a variety of circular and unilateral external fixation devices, lengthening over nails (LON), and most recently lengthening with intramedullary telescoping rods [8–11]. Each technique carries with it the potential for significant complications [12–14]; many risk factors have been identified [15].

All methods of limb lengthening rely on the process of distraction osteogenesis (DO), whereby an osteotomy is performed and the bone ends are gradually displaced apart to facilitate the growth of regenerate bone [16–19]. Several factors may influence the rate at which bone regenerates; distraction rate and bone quality may be the most important [19–23]. Although a successful procedure, problems associated with the growth of the regenerate bone have been commonly described. These difficulties may include premature consolidation, but also slow regenerate bone formation, delayed mineralization, or nonunion, which may contribute to the high rates of patient morbidity seen in limb lengthening procedures [24]. Many options, both pharmacological and non-pharmacological, have been explored to enhance the rate of growth of regenerate bone in humans and animals [25–29]. Within the non-pharmacological alternatives, studies have described the use of low intensity pulsed ultrasound (LIPUS) as well as pulsed electromagnetic fields (PEMF) during DO to enhance bone regeneration in animals [30–35]. Studies have demonstrated that LIPUS and PEMF stimulate many different cell types involved in bone healing, including osteoblasts, osteoclasts, chondrocytes, and mesenchymal cells [36]. In addition, these methods of stimulation have also been shown to increase the expression of numerous genes (including IGF and TGF-β), the size of the chondrocyte population, the synthesis of extracellular matrix, and the rate of osteoblast differentiation [37–39].

While previous studies have attempted to assess the efficacy of these alternatives in humans [40–46], there has not been a study that has systematically evaluated the success of LIPUS and PEMF in stimulating the regenerate bone growth and improving patient outcomes. Therefore, the purposes of this study were: (1) to determine the efficacy of these two non-pharmacological alternatives utilized to stimulate regenerate bone healing and (2) to establish the success rate of these alternatives in decreasing treatment time and reducing the complications during limb lengthening.

## Methods

We performed a systematic literature search to determine the possible alternatives to stimulate regenerate bone healing. This was performed utilizing the preferred reporting items for systematic review and meta-analysis protocols (PRISMA) guidelines [47]. The electronic databases Medline, Embase and Ovid were queried to find all relevant studies published in literature until July 2015. We utilized the search strings “limb lengthening,” “distraction osteogenesis,” “bone transport,” or ”regenerate bone,” which yielded a total of 16372 results. Excluding non-human studies returned 10384 publications. Further limiting the search string to studies written only in English returned 9044 studies.

**Fig. 1:**
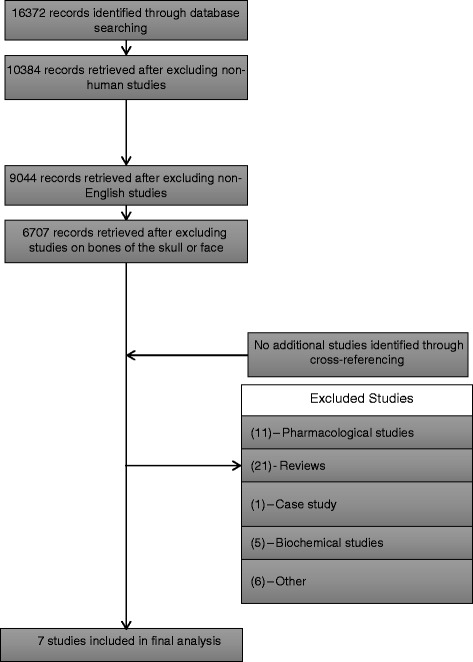
Flowchart of search methodology and inclusion/exclusion criteria

The titles and abstracts of these studies were then carefully reviewed utilizing specific inclusion and exclusion criteria. We specifically included studies evaluating the use of LIPUS or PEMF to stimulate the regenerate bone formation in limb lengthening patients. Single case reports, review studies, and literature involving maxillary or mandibular DO and craniofacial or maxillofacial surgery were excluded. After applying these inclusion and exclusion criteria, 7 studies were determined to be relevant. The citations for these studies were cross-referenced; however, no additional relevant studies were found (Fig. 1).

The entire process was performed by one of the authors (JJJ) and then fully repeated by another (AVV), blinded from the previously performed search to ensure all pertinent studies were included. We searched for specific endpoints within each study, which included age, bone (humerus, tibia, or femur) lengthened, average distraction distance, healing index, bone mineral density, and indication for bone lengthening. The information obtained from the literature review was logged into an electronic spreadsheet (Microsoft Excel, Microsoft Office, Redmond, Washington). Then, utilizing a Random Model Effects, Forest-Plots were obtained to compare the differences in bone healing index with and without the use of a bone stimulator. This was performed with the aid of statistical software (MedCalc, MedCalc Software version 15.2, Ostend, Belgium). This study was performed without external funding.

## Results

The seven studies included in our final analysis evaluated a total of 192 cases of limb lengthening and averaged 27 limbs lengthened per study [40–46]. One hundred fifty-three patients comprised of 118 males and 35 females with a mean weighted age of 26 years (range of means 8 to 39 years) underwent limb lengthening procedures. Thirty-nine of these patients underwent bilateral, symmetrical lengthening with 30 patients receiving PEMF on one of the lengthened limbs, 7 receiving LIPUS bilaterally, and 2 patients in the control group. Across all studies included in the analysis, 155 tibiae, 25 femora, and 12 humeri had undergone the index procedure (Table 1).

**Table 1:** Study demographics

Authors, Year	Number of Patients	Number of Limbs	Mean Age in Years (Range)	Tibia (%)	Femur (%)	Humerus (%)	Modality Used
Salem and Schmelz, 2014 [43]	21	21	31 (− to -)	100 %	0 %	0 %	LIPUS
El-Mowafi and Mohsen, 2005 [41]	20	20	35 (18 to 45)	100 %	0 %	0 %	LIPUS
Dudda et al., 2011 [40]	36	36	39 (16 to 69)	100 %	0 %	0 %	LIPUS
Gebauer and Correll, 2005 [42]	13	17	7.85 (− to -)	94 %	6 %	0 %	LIPUS
Gonzalez et al., 2005 [44]	30	60	11 (− to -)	47 %	33 %	20 %	PEMF
Eyres et al., 1996 [45]	13	18	17.9 (11 to 19)	78 %	22 %	0 %	PEMF
Gold and Wasserman, 2005 [46]	20	20	34 (18 to 50)	100 %	0 %	0 %	LIPUS

A total of 103 limbs were stimulated; of these, 63 were stimulated with LIPUS and 40 with PEMF. The mean amount of limbs stimulated per study was 15. Most studies applied LIPUS or PEMF concurrently during the distraction phase, with the exceptions of the studies by El-Mowafi and Mohsen [41] (LIPUS initiated one day after cessation of distraction) and Gebauer and Correll [42] (LIPUS initiated if the calcification of the newly formed bone did not improve for at least 3 months). The mean distraction distance in the treatment cohort was 8.1 cm (range of means 6.1 to 11.3 cm). For those studies reporting, the mean healing index for the treatment cohort was 33.7 days/cm ranging from 30 to 39 days/cm (Table 2). A total of 89 limbs with an average of 15 limbs per study underwent lengthening but were not stimulated. The mean distraction distance in the control cohorts was 8.2 cm (range of means 6.1 to 11.3 cm). A mean healing index of 45.4 days/cm ranging from 44 to 48 days/cm was found in those studies that reported the parameter (Table 2).

**Table 2:** Specific Outcome Measures

Authors, Year	Cohort	Number of Limbs	Mean Distraction in cm (Range)	Mean Healing Index in days/cm (± SD)	Mean Time to Fixator Removal (days) (±SD)	Mean Time to Corticalisation (days) (±SD)
Salem and Schmelz, 2014 [43]	Stimulated	12	7.9 (− to -)	33 (± −)	NR	NR
	Control	9	7.9 (− to -)	45 (± −)	NR	NR
El-Mowafi and Mohsen, 2005 [41]	Stimulated	10	6.1 (5 to 8)	30 (±2.96)	NR	NR
	Control	10	6.1 (5 to 8)	48 (±9.76)	NR	NR
Dudda et al., 2011 [40]	Stimulated	16	6.6 (2.5 to 14.0)	32.8 (±13.1)	NR	NR
	Control	20	6.6 (2.5 to 14.0)	44.4 (±6.8)	NR	NR
Gebauer and Correll, 2005 [42]	Stimulated	17	7.06 (2.5 to 14.0)	*N =* 7, calcification after 6 weeks; *N =* 10, bridging observed after 12 weeks.		
Gonzalez et al., 2005 [44]	Stimulated	30	11.3 (5.3 - 15.3)	NR	308.3 (±62.82)	279.6 (±68.4)
	Control	30	11.3 (53 to 153)	NR	339.5 (±61.17)	313.5 (±60.6)
Eyres et al., 1996 [45]	Stimulated	10	7.6 (− to -)	39 (±4)	NR	NR
	Control	8	7.6 (− to -)	44 (±6)	NR	NR
Gold and Wasserman, 2005 [46]	Stimulated	8	10.25 (8 to 14)	NR	417.3 (± −)	NR
	Control	12	9.46 (4 to 20)	NR	501.3 (± −)	NR

*NR* Not Reported

Of the 141 patients described in these studies, 70 were treated with a circular external fixation device, 64 were treated with a monolateral external fixator, and 7 were treated with hybrid fixation (unilateral and circular ring fixators) [48]. In terms of the indication for the procedure, there were 40 posttraumatic leg length discrepancies (LLD), 14 congenital LLD, 38 symmetric growth restrictions (i.e. achondroplasia), and 5 had unclassified indications (Table 3). Additionally, eight bone transports were included in our study. Only two studies described specific complications: El-Mowafi and Mohsen [41] reported 5 complications, which were 1 case of delayed union in the treatment group, 4 cases of delayed union in the control group, and 1 failure to consolidate in the control group. Dudda et al. [40] reported 6 complications, which were 1 amputation in both the treatment and control groups, 1 pseudoarthrosis in the treatment group, and 3 pseudoarthroses in the control group (Table 3). All of the other studies evaluated failed to mention complication rates.

**Table 3:** Type of fixator, indications for lengthening, and complications

		Indications for Lengthening (number)					
Authors, Year	Fixator type	Bone Transport	Post-traumatic LLD	Congenital LLD	Symmetric Growth Restrictions	Other LLD	Complications
Salem and Schmelz, 2014 [43]	Circular	0	21	0	0	0	NR
El-Mowafi and Mohsen, 2005 [41]	Circular	0	18	Congenital Anterolateral Bowing of Tibia (2)	0	0	4 delayed unions, 1 failure to consolidate
Dudda et al., 2011 [40]	Monolateral (23), Circular (6), hybrid (7)	0	NR	NR	NR	NR	2 amputations (infection), 4 Pseudoarthroses
Gebauer and Correll, 2005 [42]	Circular	0	1	Fibular hemimelia (5), CFD (1), Congenital Pseudoarthrosis (1)	Achondroplasia (3), Russell-Silver syndrome (1)	Polio (1)	NR
Gonzalez et al., 2005 [44]	Monolateral	0	0	Chondrodysplasia (1), fibular hemimelia (1)	Achondroplasia (24), Turner's Syndrome (4)	0	NR
Eyres et al., 1996 [45]	Monolateral (16), Circular (2)	0	0	Congenital short femur and tibia (2), Fibular hemimelia (1)	Achondroplasia (4), Turner's Syndrome (2)	Polio (1), infection (1), radiation (1), Ollier's (1)	NR
Gold and Wasserman, 2005 [46]	Circular	8	0	0	0	0	NR

*NR* Not Reported

**Fig. 2:**
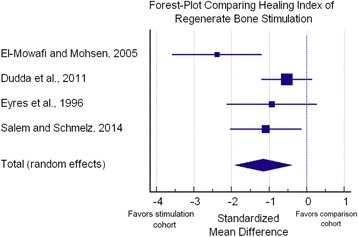
Forest-plot demonstrating the healing index between both cohorts. Forest-plot showing the effects of LIPUS or PEMF treatment on mean healing index. Each square represents the size of the study while bars represent confidence intervals. The diamond at the bottom of the graph shows the average effect size with a random effect model of the four studies; the lateral tips of the diamond represent the associated confidence interval. Note that a standardized mean difference lower than zero (0), favors stimulation (less time for bone healing)

Of the seven studies, only four studies provided detailed information regarding the differences in the healing index between the stimulated (treatment) and non-stimulated (control) cohorts. We found that the mean healing index was 11.7 days/cm faster when using bone stimulation that in the comparison cohorts (33.7 vs 45.4 days/cm), with a standardized mean difference of 1.16 (95 % Confidence Intervals of 0.40 to 1.91; *p =* 0.003), favoring a better healing index within the stimulation cohort when compared to the control cohorts (Fig. 2). Of the studies that did not provide sufficient data to incorporate into the analysis, Gonzalez et al. [44] reported a shorter time to fixator removal (308.3 vs. 339.5 days), shorter time to corticalization (279.6 vs. 313.5 days), increased callus thickness (31.2 vs. 21.8 mm), increased cortical thickness (2.73 vs. 2.63 mm), and increased bone callus density (85.7 vs. 69.8 g/cm^3^) in limbs stimulated with PEMF compared to those that were not. Similarly, Gold and Wasserman [46] found a decreased time in external fixation frame (13.91 vs. 16.71 months) and a decreased external fixation index (time in frame per cm bone transported of 1.34 vs. 2.02; Table 2). Gebauer and Correll [42] did not utilize a control group but demonstrated that LIPUS can successfully salvage delayed unions or non-unions following limb lengthening (DO).

## Discussion

Although limb lengthening has been successfully utilized to treat leg length discrepancies or short stature, the procedure is not without its inherent risks and complications [15]. Of these, many are related to slower bone healing or even non-unions. There has been a recent increase in the popularity of LIPUS in the fracture/non-union setting, and a recent meta–analysis by Rutten et al. [49] described that LIPUS was able to reduce the time to radiographic fracture healing. Similarly, in the setting of limb lengthening we found that both LIPUS and PEMF improved the healing index and decreased the amount of time needed to consolidate regenerate bone and remain in fixation. Time in fixation has been suggested to be a predictor of complications in these procedures [15]; thus, the use of LIPUS or PEMF may reduce the incidence of associated complications. It is important to note that while LIPUS and PEMF have now been demonstrated to be efficacious in both fracture healing and DO, these two processes of bone healing differ in several key ways. While fracture healing and DO both employ intramembranous and endochondral bone formation, in fracture healing endochondral bone formation is the primary method of ossification, while in DO, intramembranous ossification predominates [50, 51]. In comparable times post-injury, DO exhibits large amounts of unmineralized osteoid in the central region of the distraction gap, while fracture callus has already calcified. During fracture healing the process of angiogenesis is initiated between days 7 and 14 while in DO, angiogenesis only occurs only after active distraction commences. There are also molecular differences between the two processes: in fracture healing, IL-1, IL-6, and TNF-α are elevated soon after injury, compared to DO in which only IL-1 and IL-6 become significantly elevated [52].

There were several limitations in this study. Due to the low frequency of limb lengthening procedures, and the even rarer number of studies that have evaluated the stimulation of the regenerate bone, the overall size of the analyzed cohort is small. In addition, the studies that were included in our analysis exhibited heterogeneity in terms of mean patient age, indication for lengthening (congenital condition vs. trauma), type of bone lengthened, method of lengthening, as well as the reported outcome parameters. Hence, the overall reproducibility of our results may be limited. However, this is the first study that has comprehensively evaluated both LIPUS and PEMF to stimulate bone formation following limb lengthening. In addition, although we found a significantly faster healing time, this may not necessarily apply to the clinical and patient reported outcome measures, which require further study. Also, certain studies included in the analysis were unblinded trials, which may have introduced bias into the results, however, these studies did not receive funding from either party. While there was insufficient data to generate a funnel plot, sources of funding and competing interests for each individual study were carefully reviewed to assess for possible bias. Only one study reported a possible conflict of interest as the senior author is a consultant for the Exogen manufacturer; however, the authors received nothing of value [42].

The adjuvant properties of LIPUS and PEMF for bone healing have been known for several years. One of the first studies that evaluated regenerate bone stimulation in animals was described by Pilla et al. [53], who assessed the regenerative properties of LIPUS in fractured rabbit fibulae (*n =* 139 rabbits). They found that LIPUS-stimulated fibulae exhibited a biomechanical healing rate 1.7 times faster than that of unstimulated fibulae. Fredericks et al. [35] found that following DO, PEMF-stimulated rabbit tibiae exhibited higher mean torque-to-fracture values compared with unstimulated tibiae. Several other studies have evaluated the potentially beneficial effects of LIPUS and PEMF on fracture healing [54–59]. Heckman et al. [55] demonstrated a statistically significant decrease in the healing time of fractured tibiae treated with LIPUS as compared to untreated tibiae, while Sharrard [57] showed that PEMF stimulation of delayed unions contributed to better outcomes in stimulated tibiae compared with unstimulated tibiae.

There are also invasive alternatives that could be used to stimulate the regenerate bone, either in place of or in conjunction with the previously mentioned modalities. A study by Lee et al. [27] demonstrated that bone marrow aspirate combined with platelet rich plasma (PRP) injection following DO led to a significant improvement in the mean cortical healing indices as compared to an untreated cohort (*p <* 0.001). Similarly, Kitoh [25] showed that transplanted bone marrow cells along with PRP improved average healing indices of patients treated for short stature or LLD as compared to an untreated cohort (*p =* 0.0019 and *p =* 0.0031, respectively). These modalities have also been studied in several animal studies with successful outcomes; however, more prospective, randomized controlled trials are needed to clarify their effects [26, 28–30].

## Conclusion

Though limited by the inadequate number of studies and small number of patients in these studies, the results we obtained and the literature we reviewed support the use of LIPUS or PEMF following DO. The use of either modality improves regenerate bone formation and decreases the healing time and the amount of time spent in fixation. This may prevent complications such as delayed union, nonunion, or malunion, as well as decrease the morbidity associated with prolonged external fixation. At the current time however, the use of ultrasound is largely limited to nonunion cases. The vast majority of insurances will not reimburse for this modality unless the patient is 3 months postoperative and the affected limb remains non-united. Despite these restrictions, we believe that surgeons performing limb lengthening should consider the possibility of utilizing these non-invasive methods for regenerate bone stimulation. However, future studies with larger cohorts are needed to fully evaluate the potential success of these modalities.

## Additional files

Additional file 1: Search strategy: Search details; Contains hyperlink to the specific search string utilized for the literature search; Contains operator string utilized for the literature search. (DOCX 13 kb)
Additional file 2: Regenerate Bone Data: Pooled Data from Literature; Contains data from studies included in the analysis, including number of patients, mean age, bone segments lengthened, indications for lengthening, average distraction distance, and mean healing index. (XLSX 56 kb)

## Abbreviations

DO: Distraction osteogenesis
LIPUS: Low intensity pulsed ultrasound
LLD: Leg length discrepancy
NR: Not reported
PEMF: Pulsed electromagnetic fields

## References

[1] Manner HM, Huebl M, Radler C, Ganger R, Petje G, Grill F (2007). Accuracy of complex lower-limb deformity correction with external fixation: a comparison of the Taylor Spatial Frame with the Ilizarov ring fixator. J Child Orthop.

[2] Dammerer D, Kirschbichler K, Donnan L, Kaufmann G, Krismer M, Biedermann R (2011). Clinical value of the Taylor Spatial Frame: a comparison with the Ilizarov and Orthofix fixators. J Child Orthop.

[3] Paley D, Herzenberg JE, Paremain G, Bhave A (1997). Femoral lengthening over an intramedullary nail. A matched-case comparison with Ilizarov femoral lengthening. J Bone Joint Surg Am.

[4] Maffuli N, Fixsen JA (1996). Distraction osteogenesis in congenital limb length discrepancy: a review. J R Coll Surg Edinb.

[5] Maffulli N, Lombari C, Matarazzo L, Nele U, Pagnotta G, Fixsen JA (1996). A review of 240 patients undergoing distraction osteogenesis for congenital post-traumatic or postinfective lower limb length discrepancy. J Am Coll Surg.

[6] Codivilla A (1904). On the means of lengthening, in the lower limbs, the muscles and tissues which are shortened through deformity. Clin Orthop Relat Res.

[7] Ilizarov GA (1990). Clinical application of the tension-stress effect for limb lengthening. Clin Orthop Relat Res.

[8] Paley D (1988). Current techniques of limb lengthening. J Pediatr Orthop.

[9] Chaudhary M (2008). Limb lengthening over a nail can safely reduce the duration of external fixation. Indian J Orthop.

[10] Paley D (2015). PRECICE intramedullary limb lengthening system. Expert Rev Med Devices.

[11] Rozbruch SR, Kleinman D, Fragomen AT, Ilizarov S (2008). Limb lengthening and then insertion of an intramedullary nail: a case-matched comparison. Clin Orthop Relat Res.

[12] Eldridge JC, Bell DF (1991). Problems with substantial limb lengthening. Orthop Clin North Am.

[13] Dahl MT, Gulli B, Berg T (1994). Complications of limb lengthening. A learning curve Clinical orthopaedics and related research.

[14] Hantes ME, Malizos KN, Xenakis TA, Beris AE, Mavrodontidis AN, Soucacos PN (2001). Complications in limb-lengthening procedures: a review of 49 cases. Am J Orthop (Belle Mead NJ).

[15] Liantis P, Mavrogenis AF, Stavropoulos NA, Kanellopoulos AD, Papagelopoulos PJ, Soucacos PN, Babis GC (2014). Risk factors for and complications of distraction osteogenesis. Eur J Orthop Surg Traumatol.

[16] Hasler CC, Krieg AH (2012). Current concepts of leg lengthening. J Child Orthop.

[17] De Bastiani G, Aldegheri R, Renzi-Brivio L, Trivella G (1987). Limb lengthening by callus distraction (callotasis). J Pediatr Orthop.

[18] Ilizarov GA (1989). **The tension-stress effect on the genesis and growth of tissues.** Part I. The influence of stability of fixation and soft-tissue preservation. Clin Orthop Relat Res.

[19] Ilizarov GA (1989). **The tension-stress effect on the genesis and growth of tissues: Part II.** The influence of the rate and frequency of distraction. Clin Orthop Relat Res.

[20] Fink B, Krieger M, Strauss JM, Opheys C, Menkhaus S, Fischer J, Ruther W (1996). Osteoneogenesis and its influencing factors during treatment with the Ilizarov method. Clin Orthop Relat Res.

[21] White SH, Kenwright J (1990). The timing of distraction of an osteotomy. J Bone Joint Surg.

[22] Yasui N, Kojimoto H, Sasaki K, Kitada A, Shimizu H, Shimomura Y (1993). Factors affecting callus distraction in limb lengthening. Clin Orthop Relat Res.

[23] Fischgrund J, Paley D, Suter C (1994). Variables affecting time to bone healing during limb lengthening. Clin Orthop Relat Res.

[24] Paley D (1990). Problems, obstacles, and complications of limb lengthening by the Ilizarov technique. Clin Orthop Relat Res.

[25] Kitoh H, Kitakoji T, Tsuchiya H, Katoh M, Ishiguro N (2007). Transplantation of culture expanded bone marrow cells and platelet rich plasma in distraction osteogenesis of the long bones. Bone.

[26] Raschke MJ, Bail H, Windhagen HJ, Kolbeck SF, Weiler A, Raun K, Kappelgard A, Skiaerbaek C, Haas NP (1999). Recombinant growth hormone accelerates bone regenerate consolidation in distraction osteogenesis. Bone.

[27] Lee DH, Ryu KJ, Kim JW, Kang KC, Choi YR (2014). Bone marrow aspirate concentrate and platelet-rich plasma enhanced bone healing in distraction osteogenesis of the tibia. Clin Orthop Relat Res.

[28] Yang JH, Kim HJ, Kim SE, Yun YP, Bae JH, Kim SJ, Choi KH, Song HR (2012). The effect of bone morphogenic protein-2-coated tri-calcium phosphate/hydroxyapatite on new bone formation in a rat model of femoral distraction osteogenesis. Cytotherapy.

[29] Zhu S, Song D, Jiang X, Zhou H, Hu J (2011). Combined effects of recombinant human BMP-2 and Nell-1 on bone regeneration in rapid distraction osteogenesis of rabbit tibia. Injury.

[30] Shimazaki A, Inui K, Azuma Y, Nishimura N, Yamano Y (2000). Low-intensity pulsed ultrasound accelerates bone maturation in distraction osteogenesis in rabbits. J Bone Joint Surg.

[31] Tis JE, Meffert CR, Inoue N, McCarthy EF, Machen MS, McHale KA, Chao EY (2002). The effect of low intensity pulsed ultrasound applied to rabbit tibiae during the consolidation phase of distraction osteogenesis. J Orthopaedic res.

[32] Claes L, Ruter A, Mayr E (2005). Low-intensity ultrasound enhances maturation of callus after segmental transport. Clin Orthop Relat Res.

[33] Eberson CP, Hogan KA, Moore DC, Ehrlich MG (2003). Effect of low-intensity ultrasound stimulation on consolidation of the regenerate zone in a rat model of distraction osteogenesis. J Pediatr Orthop.

[34] Taylor KF, Inoue N, Rafiee B, Tis JE, McHale KA, Chao EY (2006). Effect of pulsed electromagnetic fields on maturation of regenerate bone in a rabbit limb lengthening model. J Orthopaedic Res.

[35] Fredericks DC, Piehl DJ, Baker JT, Abbott J, Nepola JV (2003). Effects of pulsed electromagnetic field stimulation on distraction osteogenesis in the rabbit tibial leg lengthening model. J Pediatr Orthop.

[36] Claes L, Willie B (2007). The enhancement of bone regeneration by ultrasound. Prog Biophys Mol Biol.

[37] Hannouche D, Petite H, Sedel L (2001). Current trends in the enhancement of fracture healing. J Bone Joint Surg.

[38] Aaron RK, Ciombor DM (1996). Acceleration of experimental endochondral ossification by biophysical stimulation of the progenitor cell pool. J Orthopaedic Res.

[39] Sollazzo V, Palmieri A, Pezzetti F, Massari L, Carinci F (2010). Effects of pulsed electromagnetic fields on human osteoblastlike cells (MG-63): a pilot study. Clin Orthop Relat Res.

[40] Dudda M, Hauser J, Muhr G, Esenwein SA (2011). Low-intensity pulsed ultrasound as a useful adjuvant during distraction osteogenesis: a prospective, randomized controlled trial. J Trauma.

[41] El-Mowafi H, Mohsen M (2005). The effect of low-intensity pulsed ultrasound on callus maturation in tibial distraction osteogenesis. Int Orthop.

[42] Gebauer D, Correll J (2005). Pulsed low-intensity ultrasound: a new salvage procedure for delayed unions and nonunions after leg lengthening in children. J Pediatr Orthop.

[43] Salem KH, Schmelz A (2014). Low-intensity pulsed ultrasound shortens the treatment time in tibial distraction osteogenesis. Int Orthop.

[44] Luna Gonzalez F, Lopez Arevalo R, Meschian Coretti S, Urbano Labajos V, Delgado Rufino B (2005). Pulsed electromagnetic stimulation of regenerate bone in lengthening procedures. Acta Orthop Belg.

[45] Eyres KS, Saleh M, Kanis JA (1996). Effect of pulsed electromagnetic fields on bone formation and bone loss during limb lengthening. Bone.

[46] Gold SM, Wasserman R (2005). Preliminary results of tibial bone transports with pulsed low intensity ultrasound (Exogen). J Orthop Trauma.

[47] Shamseer L, Moher D, Clarke M, Ghersi D, Liberati A, Petticrew M, Shekelle P, Stewart LA, Group P-P (2015). Preferred reporting items for systematic review and meta-analysis protocols (PRISMA-P) 2015: elaboration and explanation. BMJ.

[48] Fragomen AT, Rozbruch SR (2007). The mechanics of external fixation. HSS J.

[49] Rutten S, van den Bekerom MP, Sierevelt IN, Nolte PA (2016). Enhancement of Bone-Healing by Low-Intensity Pulsed Ultrasound: A Systematic Review. JBJS Rev.

[50] Yasui N, Sato M, Ochi T, Kimura T, Kawahata H, Kitamura Y, Nomura S (1997). Three modes of ossification during distraction osteogenesis in the rat. J Bone Joint Surg.

[51] Einhorn TA. The cell and molecular biology of fracture healing. Clin Orthop Relat Res. 1998;(355 Suppl):S7-21. PubMed PMID: 9917622. 10.1097/00003086-199810001-00003 9917622

[52] Ai-Aql ZS, Alagl AS, Graves DT, Gerstenfeld LC, Einhorn TA (2008). Molecular mechanisms controlling bone formation during fracture healing and distraction osteogenesis. J Dent Res.

[53] Pilla AA, Mont MA, Nasser PR, Khan SA, Figueiredo M, Kaufman JJ, Siffert RS (1990). Non-invasive low-intensity pulsed ultrasound accelerates bone healing in the rabbit. J Orthop Trauma.

[54] Rubin C, Bolander M, Ryaby JP, Hadjiargyrou M (2001). The use of low-intensity ultrasound to accelerate the healing of fractures. J Bone Joint Surg Am.

[55] Heckman JD, Ryaby JP, McCabe J, Frey JJ, Kilcoyne RF (1994). Acceleration of tibial fracture-healing by non-invasive, low-intensity pulsed ultrasound. J Bone Joint Surg Am.

[56] Kristiansen TK, Ryaby JP, McCabe J, Frey JJ, Roe LR (1997). Accelerated healing of distal radial fractures with the use of specific, low-intensity ultrasound. A multicenter, prospective, randomized, double-blind, placebo-controlled study. J Bone Joint Surg Am.

[57] Sharrard WJ (1990). A double-blind trial of pulsed electromagnetic fields for delayed union of tibial fractures. J Bone Joint Surg.

[58] Simonis RB, Parnell EJ, Ray PS, Peacock JL (2003). Electrical treatment of tibial non-union: a prospective, randomised, double-blind trial. Injury.

[59] Barker AT, Dixon RA, Sharrard WJ, Sutcliffe ML (1984). Pulsed magnetic field therapy for tibial non-union. Interim results of a double-blind trial. Lancet.

