# Supplementary material for: Regenerate bone stimulation following limb lengthening: a meta-analysis
Source: BMC Musculoskelet Disord. 2016 Sep 29;17:407. doi: 10.1186/s12891-016-1259-5 (PMC5043605; doi:10.1186/s12891-016-1259-5)
Supplement: Additional file 1: — Search strategy: Search details; Contains hyperlink to the specific search string utilized for the literature search; Contains operator string utilized for the literature search. (DOCX 13 kb) [file 12891_2016_1259_MOESM1_ESM.docx]

**Search Strategy**

**Link of Search String:**

<http://www.ncbi.nlm.nih.gov/pubmed/?term=regenerate+bone+OR+limb+lengthening+OR+distraction+osteogenesis+OR+bone+transport+NOT+(cranio-+OR+craniofacial+OR+maxillo-+OR+maxillary+OR+maxillofacial-+OR+mandibular-+OR+temporomandibular+OR+facial+OR+skull+OR+face+OR+cleft+palate)>

**Specific search details:**

(regenerate[All Fields] AND ("bone and bones"[MeSH Terms] OR ("bone"[All Fields] AND "bones"[All Fields]) OR "bone and bones"[All Fields] OR "bone"[All Fields])) OR (("extremities"[MeSH Terms] OR "extremities"[All Fields] OR "limb"[All Fields]) AND lengthening[All Fields]) OR ("osteogenesis, distraction"[MeSH Terms] OR ("osteogenesis"[All Fields] AND "distraction"[All Fields]) OR "distraction osteogenesis"[All Fields] OR ("distraction"[All Fields] AND "osteogenesis"[All Fields])) OR (("bone and bones"[MeSH Terms] OR ("bone"[All Fields] AND "bones"[All Fields]) OR "bone and bones"[All Fields] OR "bone"[All Fields]) AND ("biological transport"[MeSH Terms] OR ("biological"[All Fields] AND "transport"[All Fields]) OR "biological transport"[All Fields] OR "transport"[All Fields])) NOT (("Cranio"[Journal] OR "cranio"[All Fields]) OR craniofacial[All Fields] OR maxillo-[All Fields] OR ("maxilla"[MeSH Terms] OR "maxilla"[All Fields] OR "maxillary"[All Fields]) OR maxillofacial-[All Fields] OR ("mandible"[MeSH Terms] OR "mandible"[All Fields] OR "mandibular"[All Fields]) OR temporomandibular[All Fields] OR ("face"[MeSH Terms] OR "face"[All Fields] OR "facial"[All Fields]) OR ("skull"[MeSH Terms] OR "skull"[All Fields]) OR ("face"[MeSH Terms] OR "face"[All Fields]) OR ("cleft palate"[MeSH Terms] OR ("cleft"[All Fields] AND "palate"[All Fields]) OR "cleft palate"[All Fields])) AND (("0001/01/01"[PDAT] : "2015/06/30"[PDAT]) AND "humans"[MeSH Terms] AND English[lang])
